# DISCRIMINATION AND RESILIENCE ON HEALTH ACROSS THE LIFESPAN

**DOI:** 10.1093/geroni/igae098.3246

**Published:** 2024-12-31

**Authors:** Yeonjung Lee, Cindy Vang, Youhung Her-Xiong

**Affiliations:** University of Hawaiʻi at Mānoa, Honolulu, Hawaii, United States; California State University Chico, Chico, California, United States; Q&C Research Consulting, LLC, Eau Claire, Wisconsin, United States

## Abstract

Asian and Asian American (AAA) older adults have encountered varying forms of discrimination in the United States. However, discrimination across the life course shaping the lives of AAA is an emerging area in understanding health disparities. This study aimed to shed light on this area by using the Life Course Perspective and the Asian Critical Theory to characterize the levels of discrimination, resilience, and health of AAA older adults before and after the COVID-19 pandemic. The preliminary data were collected through convenience and snowball sampling methods. Participants (N=39) completed a survey using discrimination, resilience, and health measures before and during the pandemic. Descriptive results indicated increased perceived mild discrimination post-pandemic compared to the pre-COVID-19 pandemic. The findings, however, indicate that participants showed increased resilience during the COVID-19 pandemic. Based on the preliminary data focusing on hard-to-reach older adults, the present study findings can help understand the unique association between discrimination and resilience experiences among AAA older adults.

